# Variation in Copy Number of Ty3/Gypsy Centromeric Retrotransposons in the Genomes of *Thinopyrum intermedium* and Its Diploid Progenitors

**DOI:** 10.1371/journal.pone.0154241

**Published:** 2016-04-27

**Authors:** Mikhail G. Divashuk, Thi Mai L. Khuat, Pavel Yu. Kroupin, Ilya V. Kirov, Dmitry V. Romanov, Anna V. Kiseleva, Ludmila I. Khrustaleva, Dmitry G. Alexeev, Alexandr S. Zelenin, Marina V. Klimushina, Olga V. Razumova, Gennady I. Karlov

**Affiliations:** 1 Centre for Molecular Biotechnology, Russian State Agrarian University–Moscow Timiryazev Agricultural Academy, Timiryazevskaya St. 49, 127550, Moscow, Russia; 2 Federal Research and Clinical Centre of Physical-Chemical Medicine (FRCC PCM), Moscow, Russia; Ben-Gurion University, ISRAEL

## Abstract

Speciation and allopolyploidization in cereals may be accompanied by dramatic changes in abundance of centromeric repeated transposable elements. Here we demonstrate that the reverse transcriptase part of Ty3/gypsy centromeric retrotransposon (RT-CR) is highly conservative in the segmental hexaploid *Thinopyrum intermedium* (J^r^J^vs^St) and its possible diploid progenitors *Th*. *bessarabicum* (J^b^), *Pseudoroegneria spicata* (St) and *Dasypyrum villosum* (V) but the abundance of the repeats varied to a large extent. Fluorescence *in situ* hybridization (FISH) showed hybridization signals in centromeric region of all chromosomes in the studied species, although the intensity of the signals drastically differed. In *Th*. *intermedium*, the strongest signal of RT-CR probe was detected on the chromosomes of J^v^, intermediate on J^r^ and faint on J^s^ and St subgenome suggesting different abundance of RT-CR on the individual chromosomes rather than the sequence specificity of RT-CRs of the subgenomes. RT-CR quantification using real-time PCR revealed that its content per genome in *Th*. *bessarabicum* is ~ 2 times and *P*. *spicata* is ~ 1,5 times higher than in genome of *D*. *villosum*. The possible burst of Ty3/gypsy centromeric retrotransposon in *Th*. *intermedium* during allopolyploidization and its role in proper mitotic and meiotic chromosome behavior in a nascent allopolyploid is discussed.

## Introduction

Centromeres are responsible for kinetochore assembly that links chromosome to microtubes and thus play a key role in equal chromosome segregation and transmission during cell division. In plants, the centromere sequences are usually represented by DNA satellites interspersed with long terminal repeat (LTR) centromeric retrotransposons (CR), both are associated with CENH3, centromere specific variant of histone H3 [1–7]. CRs were found as highly conserved centromere-specific sequences in grasses [2, 3] and are represented by Cereba in barley, CRW in wheat, CRR in rice, CRM in maize, CRBd1 in *Brachypodium distachyon* [3–5, 8–11]. As revealed by analysis of the reverse transcriptase (RT) sequences, CRs belong to chromoviruses (Chromoviridae), a lineage of Ty3/gypsy retrotransposons forming a phylogenetically distinct clade designated CRM [12–15].

The copy number of centromeric repeats varies among different species and even ecotypes [16–18]. The abundance of centromeric repeat may be associated with the ploidy level and their amplification and transposition may occur in response to polyploidization event [19]. In bread wheat (*Triticum aestivum*), a recently formed polyploid (BBAADD), two centromere-specific elements have been found: CRW (also called Cereba) and Quinta that are the youngest elements at the centromeres of common wheat and its diploid ancestors. It was shown that the D subgenome chromosomes contained fewer copies of CRW than either the A or B subgenome chromosomes [18].

The close relative of wheat is intermediate wheatgrass (*Thinopyrum intermedium* (Host) Barkworth & D.R. Dewey), an important forage crop and a valuable source of genes used for wheat improvement through wide hybridization [20, 21]. *Thinopyrum intermedium* is a segmental allohexpaloid (2n = 42) with complex genomic constitution. The following symbols were proposed for the designation of *Th*. *intermedium* subgenomes: J^r^ (= J = E), J^vs^ (= J^s^ = (V-J-R)^s^ = (V-J-H)^s^), and St (= S = X) [22–31] with J related to *Th*. *bessarabicum* (J^b^), St to *Pseudoroegneria spicata* (St) and V to *Dasysyprum villosum* (V). The St subgenome originated from Pseudoroegneria diploid species has been found unequivocally present in *Th*. *intermedium* [32]. The J^r^ (= J = E) and J^vs^ (= J^s^ = (V-J-R)^s^ = (V-J-H)^s^) are closely related subgenomes and assumed to be the progenitor genomes in the lineages of *Th*. *bessarabicum* (J^b^) and *Th*. *elongatum* (J^e^). The presence of repetitive sequences from V (Dasypyrum) and R (Secale) genomes in the *Th*. *intermedium* genome has been revealed by fluorescent *in situ* hybridization (FISH) [32]. Lu et al. [33] and Li et al. [34] studied chromosome distribution of the CRW (Cereba)-like elements in the genomes of *Th*. *intermedium* and related species. However, their studies did not contain comparison in the abundance of the elements between related species.

For the quantification of transposable elements (TE) in genome along with FISH, Southern hybridization, and dot blot analyses SYBR Green-based real-time quantitative PCR (qPCR) assay is applied [35, 36]. Using qPCR assay the dynamics and variation of the TE copy number has been shown in Aegilops and Triticum species [35–37]. However, the abundance of CRs using qPCR assay has not been studied in other Triticeae species so far.

Here, we characterize the reverse-transcriptase parts of the Ty3/gypsy centromeric retrotransposon (RT-CR) and demonstrate their abundance in *Th*. *intermedium*, *Th*. *bessarabicum*, *P*. *spicata* and *D*. *villosum*. The results provide valuable information to shed more light on the evolution and speciation in Triticeae.

## Results

### Sequencing and clusterization of RT-CR sequences

PCR with the TAIDF/R primers using genomic DNA of *Th*. *intermedium*, *Th*. *bessarabicum*, *P*. *spicata* and *D*. *villosum* resulted in the amplification of a pool of closely related ~ 490 bp fragments of the reverse transcriptase gene of centromeric retrotransposons (RT-CR). The PCR products were subsequently cloned and at least 20 clones were randomly selected for sequence analysis from each species. While some clones contained identical sequences only unique sequences were published in GenBank (accessions KR873398-KR873420). Homology-based searching (NCBI BLASTn and Conservative Domain Search) confirmed the similarity of the isolated sequences to the RT domains of the published centromeric RT-CR Ty3/gypsy retrotransposons of other species. The multiple alignment of the cloned RT-CR sequences revealed that they are highly homologous between each other (S1 Fig).

### Phylogenetic relationships

Phylogenetic relationships were established using nucleotide and deduced amino acid sequences. The dendrogram from DNA sequences was generated by aligning the cloned RT-CR nucleotide sequences (numbers of RT-CR SNP variants: *Th*. *bessarabicum*– 4, *Th*. *intermedium*– 9, *P*. *spicata*– 5, and *D*. *villosum*– 6) with 964 homologous to them sequences from GenBank found using NCBI BLAST search (Nucleotide collection). All RT-CRs sequences of *Th*. *intermedium*, *Th*. *bessarabicum*, *D*. *villosum* and one sequence of *P*. *spicata* shared a cluster with *T*. *aestivum*, *H*. *vulgare*, and *S*. *cereale* (S2 Fig, group 1); another cluster was shared by three RT-CRs of *P*. *spicata* together with *Z*. *mays* and *O*. *sativa* (S2 Fig, group 2). Among RT-CR sequences found using NCBI Nucleotide collection search database a great majority (632) belong to *Triticum aestivum* chromosome 3B cultivar *Chinese Spring* and was represented both in group 1 (*Th*. *intermedium*, *Th*. *bessarabicum*, *D*. *villosum* and one sequence of *P*. *spicata*) and group 2 (*P*. *spicata*) (S2 Fig).

**Fig 1:**
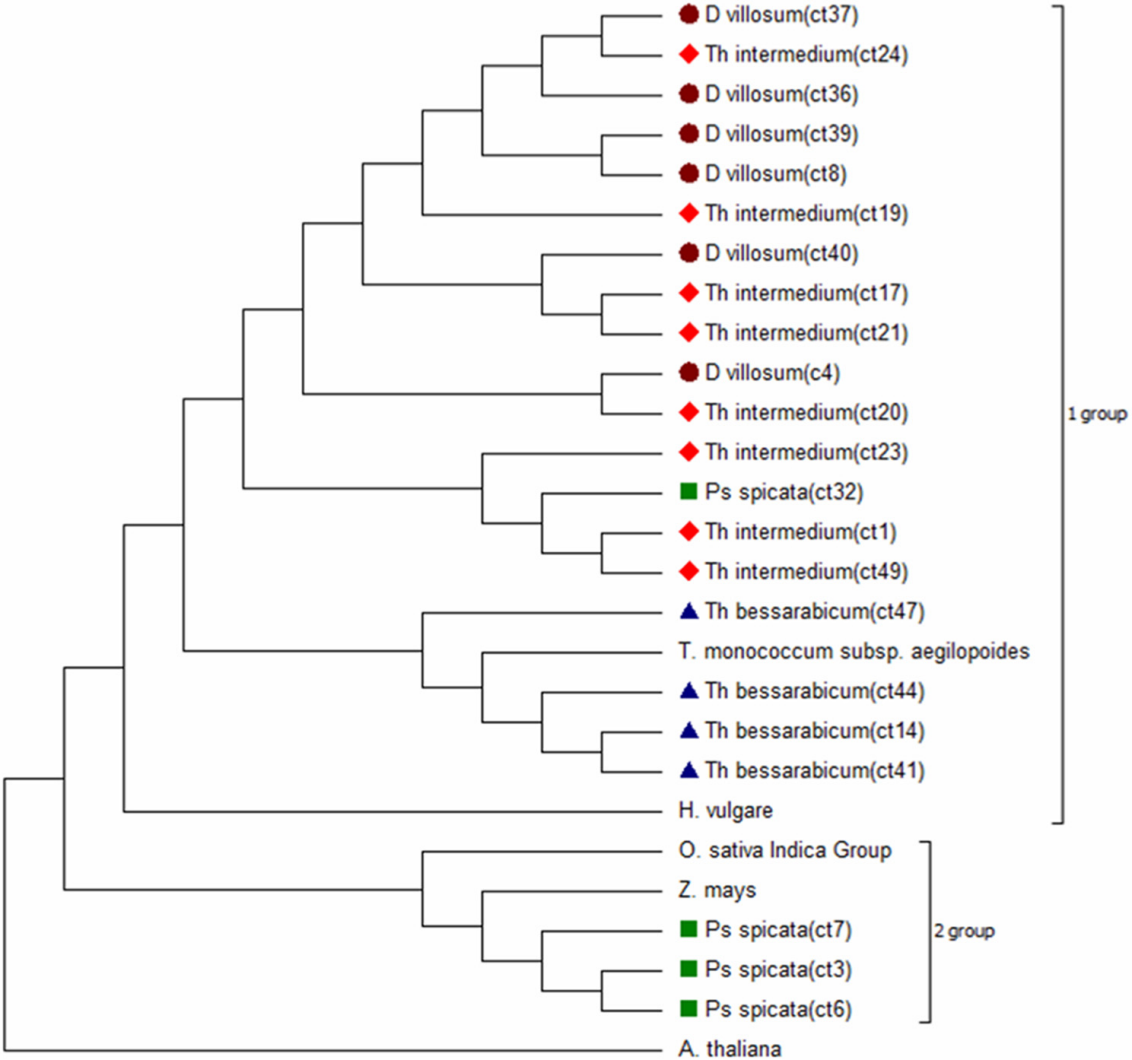
The dendrogram of the deduced RT-CR amino acid sequences and their homologs from the NCBI Nucleotide collection search database. The dendrogram was constructed using the Maximum Likelihood method based on the JTT matrix-based model. The bootstrap consensus tree inferred from 1000 replicates. The colored bullets indicate the RT-CRs obtained in the present study.

Thereafter, the phylogenetic tree from amino acid sequences was constructed by aligning RT-CR sequences obtained in this study with the RT domains of the known centromeric repeats of rice (DQ458289.1), maize (AAM94350.1), wheat (ABI96971.1) and barley (AAK94517.1) (Fig 1). The RT-CR element of *A*. *thaliana* (AAF79348.1) was used as an out-group. The RT-CRs of *Th*. *intermedium*, *Th*. *bessarabicum*, *D*. *villosum* and one of *P*. *spicata* formed one clade with RT sequences of wheat (CRW) and barley (Cereba) (Fig 1, group 1). Three sequences of the *P*. *spicata* RT-CR were more distant from others and formed their own clade with rice (CRR) and maize (CRM) (Fig 1, group 2).

Thus, in both cases the dendrograms of amino and nucleic acid sequences show similar pattern of clusterization of our RT-CRs and RT-CR sequences from wheat, rye, barley and maize.

### FISH signal on different genomes

**Fig 2:**
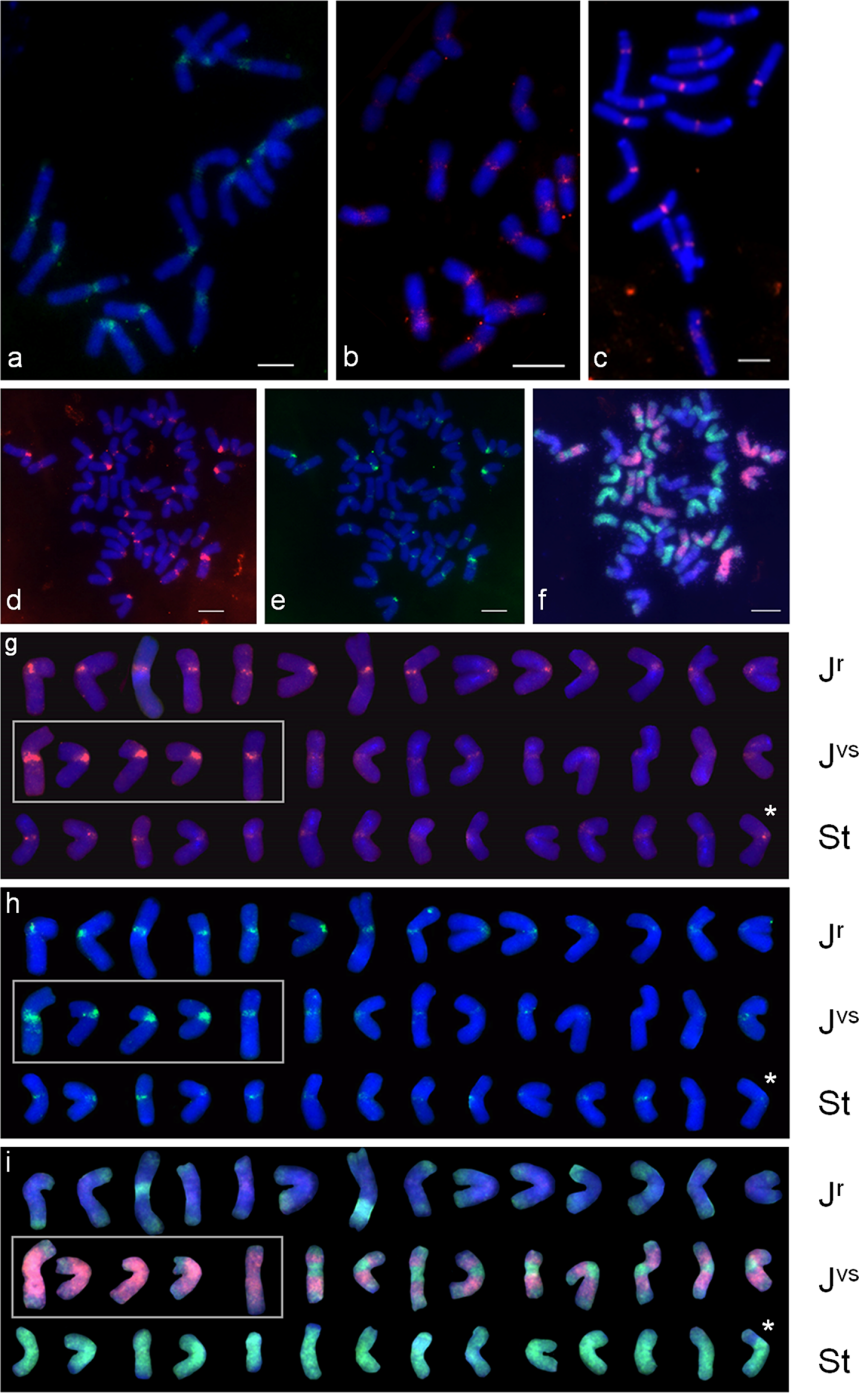
Hybridization of the RT-CR probes on chromosomes of *D*. *villosum*, *P*. *spicata*, *Th*. *bessarabicum*, and *Th*. *intermedium*. **a-c** FISH analysis using RT-CR probes on the root-tip cells at mitotic metaphase in a) *D*. *villosum*, b) *P*. *spicata*, c) *Th*. *bessarabicum*; the metaphase plates are probed with *Davi* (a, green), *Pssp* (b, pink) and *Thbe* (c, pink). Bar = 5 μm. **d-f** Sequential multicolor FISH and multicolor GISH on the root-tip cells at mitotic metaphase in *Th*. *intermedium*: d) the mcFISH results using the *Thbe* probe (red); e) the same cell in (d) with *Pssp* probe (green); f) GISH analysis for the same cell in (d) and (e) with the labeled genomic DNA of *P*. *spicata* (green) and *D*. *villosum* (pink) as probes and *T*. *aestivum* genomic DNA (ABD genome) as a block. Bar = 10 μm. **g-i** Karyotype of chromosomes of *Th*. *intermedium* from (d-f) with the results of sequential multicolor FISH and multicolor GISH. Chromosomes organized into genomes J^r^, J^vs^ and St according to St- and V-genome DNA labeling and FISH signal intensity in centromeric region. The J^vs^ chromosomes grouped into J^v^ chromosomes (in frame) and J^s^ chromosomes. The translocated chromosome marked with asterisk. g) Chromosomes with *Thbe* probe (red); h) The same chromosomes with *Pssp* probe (green); i) The same chromosomes labeled with genomic DNA of *P*. *spicata* (St genome, green) and *D*. *villosum* (V genome, pink) as probes and *T*. *aestivum* genomic DNA (ABD genome) as a block. Chromosomes counterstained with DAPI (blue).

To physically localize RT-CRs FISH was performed on the chromosomes of *Th*. *bessarabicum*, *P*. *spicata*, *D*. *villosum*, and *Th*. *intermedium* using the labeled PCR products obtained with TAID_F/R primers and the DNA of the corresponding species as a template. The PCR probes obtained from genomic DNA of *Th*. *intermedium*, *P*. *spicata*, *D*. *villosum* and *Th*. *bessarabicum* hereinafter are referred to as *Thin*, *Pssp*, *Davi* and *Thbe*, respectively. FISH resulted in clear signals located in the centromeric regions of all chromosomes of the studied species (Fig 2).

In diploid species, although slightly varying between individual chromosomes within some metaphase plates, the signal intensity was uniformly bright on all chromosomes (Fig 2A, 2B and 2C). In *Th*. *intermedium*, the *Thin* probe hybridized to the centromeres of all chromosomes but the intensity of the FISH signals varied strongly among individual chromosomes within metaphase plates. Such differences were observed on all the analyzed metaphase plates. The strongest signals were observed on five chromosomes thereby visually distinguished from other chromosomes of the complement. The hardly visible signals were detected on eight chromosomes. The remained 29 chromosomes of *Th*. *intermedium* possessed FISH signals with the intermediate intensity.

The question appeared whether the differences in signal intensity in *Th*. *intermedium* are due to the (sub)genomic specificity of the probe used (for example, due to the presence of (sub)genome specific sequences) or it is a result of different RT-CR abundance on the chromosomes. To answer the question a cross multicolor FISH was performed on the metaphase plates of *Th*. *intermedium* with the probes *Thbe*, *Pssp*, and *Davi*. If all probes hybridized specifically to the same subgenomes from which they were obtained, this would mean that they were genome-specific. In case if the sample obtained by PCR with DNA from different species with the same fluorescence intensity would hybridize to the same chromosomes of Th. intermedium metaphase plates—this would mean that the difference was in the copy number of RT-CR in different chromosomes. Three multicolor FISH experiments were performed on the *Th*. *intermedium* chromosomes using simultaneously two probes, namely: i) *Pssp* and *Thbe* (Fig 2D and 2E); ii) *Davi* and *Thbe* (data not shown); iii) *Davi* and *Pssp* (data not shown). In all FISH experiments both probes demonstrated the same chromosomal pattern of hybridization (strong and faint signals on particular chromosomes, Fig 2D and 2E) and the intensity of FISH signal on individual chromosomes did not depend on the probes used.

To identify the chromosome sets belonged to each subgenome of *Th*. *intermedium* sequential FISH/multicolor GISH (mcGISH) was carried out on the same metaphase plates. McGISH analysis on mitotic chromosomes of *Th*. *intermedium* (Fig 2F) distinguished 14 chromosomes of J^r^ subgenome (blue with green subtelomeric signals), St subgenome (green, one of which with translocation) and J^vs^ subgenome (pink with green subtelomeric and/or pericentromeric signals). Five chromosomes of J^vs^ subgenome demonstrated green subtelomeric signal localization (designated J^v^) while nine with both subtelomeric and pericentromeric signal localization (designated J^s^) (Fig 2F and 2I). Thus, five J^v^- and nine J^s^-chromosomes were identified in *Th*. *intermedium* chromosome set. In previous studies, the number of J^s^ chromosomes varied from eight to ten in *Th*. *intermedium* [23, 27, 38, 39]. We also found one St chromosome with translocation in the short arm from J^r^ chromosome (Fig 2I, asterisk). Previously, Kishii et al. [23] and Mahelka et al. [39] reported about translocation between chromosomes of different *Th*. *intermedium* subgenomes. These results suggest that the stabilization of the allopolyploid genome of *Th*. *intermedium* is still an ongoing process.

The comparison of FISH and GISH results showed that the strongest FISH signals from RT-CR PCR probe are located on all J^v^ chromosomes while the faintest signals were detected on four St chromosomes and four J^s^ chromosomes. Other chromosomes carried the signal of intermediate intensity (Fig 2G and 2H).

On average, the total signal intensity of RT-CR probe on the *Th*. *intermedium* subgenomes can be ranged as follows: J^v^>J^r^> {J^s^≈St}.

### qPCR

We performed qPCR with the primers TAID_F /R on the genomic DNA of *Th*. *bessarabicum*, *P*. *spicata*, and *D*. *villosum* to assess the relative quantity of RT-CR sequences. The analysis revealed that RT-CR content per genome in *Th*. *bessarabicum* is ~2 times and *P*. *spicata* is ~1,5 times higher than in genome of *D*. *villosum*.

Based on the results of qPCR analysis the genomes can be ranged according to the abundance of RT-CR as follows: J^b^ > St > V.

## Discussion

The activation of CRs in *Th*. *intermedium* (J^r^J^vs^St) might have happened after or as a result of allopolyploidization event. Although polyploidy-induced transposition burst of TE in plants is not a general rule it can be TE family- and species-specific and apparently fasten genome evolution in the long term [37, 40, 41]. In our study, qPCR revealed that *D*. *villosum* (V, related to J^vs^ subgenome) has significantly less copies of Ty3/gypsy CR than candidate donors for J^r^ (*Th*. *Bessarabicum*, J^b^) and St (*P*. *spicata*, St) subgenomes. These results differ from those that could be expected based on the FISH results obtained for chromosomes of each subgenome of *Th*. *intermedium*, namely, that four J^v^ chromosomes are more abundant in CRs in centromeres than other chromosomes. Assuming that *D*. *villosum*, *Th*. *bessarabicum*, *P*. *spicata* are the donors for J^vs^, J^r^ and St subgenomes of *Th*. *intermedium* (or very close to them), respectively, the observed differences in centromeric FISH signal intensity between chromosomes of different subgenomes in *Th*. *intermedium* cannot be explained by the differences in CR abundance in the genomes of donor species. Such difference may be associated with CR burst after polyploidization as it was shown in some Triticeae. Yakov et al. showed evolutionary- and revolutionary-scale increase in quantity of certain TEs in *T*. *durum* and *T*. *aestivum* in comparison to its candidate subgenome donors *Ae*. *speltoides*, *Ae*. *taushcii*, *Ae*. *searsii* and *T*. *urartu* [37]. In wheat, it was found that the time of burst of centromeric retrotransposons CRW and Quinta coincides with the time of the origin of tetraploid wheat [19, 42–44].

Although according to qPCR experiment in present study *D*. *villosum* contains the least amount of CRs, the polymorphism between different populations of diploids as well as that the absence of knowledge about the exact subgenome donors of *Th*. *intermedium* should be taken into consideration. Therefore, the alternative hypothesis also should be considered: the progenitor of J^v^ chromosomes had the CRs burst before polyploidization and its high abundance might have been a necessary condition for J^v^ to be incorporated into a newly formed polyploid genome and ensured its meiotic stability.

The stability of *Th*. *intermedium* as an allopolyploid may be associated with the expansion of CRs in the centromeric region of J^v^ chromosomes and recombinant nature of J^s^ chromosomes. Centromeric and pericentromereic regions of the chromosomes are critical areas for the differentiation of subgenomes in polyploids during meiosis [45–48]. In *Th*. *intermedium* as an allopolyploid, the proper meiotic and mitotic behavior of chromosomes is crucial for its existence as a species. Our subsequent FISH/GISH experiments showed that the strong FISH signal belongs to all J^v^ chromosomes while the faintest signals were detected on four St chromosomes and four J^s^ chromosomes. We can suggest that the expanded CR region in J^v^ chromosomes and recombined St-specific pericentromeric region in J^s^ chromosomes prevents them from pairing with homologous chromosomes of St and J^r^ subgenomes in meiosis. Notably, the signal on J^r^ chromosomes on average was stronger than on St chromosomes that also may contribute to certain extent to their differentiation at meiosis. It was shown that genomic changes resulted from TE transposition may impede the homeologous chromosome pairing facilitating homologous pairing during meiosis [49, 50]. Similarly, Liu et al. showed that in *T*. *aestivum* that D subgenome chromosomes contained fewer copies of CRW than either the A or B subgenome chromosomes [18]. In our sequential GISH/FISH experiments on *T*. *aestivum* cv. Tulaykovskaya 100 (with 6J^r^(6D) substitution [51]), the probes *Thbe*, *Thin*, *Pssp*, and *Davi* hybridized to the centromeric regions and the observed variation in FISH signal intensity among *T*. *aestivum* chromosomes was not as sharp as in *Th*. *intermedium*, the centromeric signal in 6J^r^ was fainter than in wheat chromosomes (S3 Fig). Therefore, the variation in CR abundance between *Th*. *intermedium* subgenomes exceeds that in wheat.

Based on the results of the analysis of nucleic acid and deduced amino acid sequences of RT-CR, we propose that there might have been a pool of different CR sequences in ancestral species. During speciation some of them predominantly amplified in different species while copy number of other RT variants did not undergo significant changes. This assumption is based on that although RT-CR variants of the studied species were included into two separate clades, the sequences of *T*. *aestivum* chromosome 3B were represented in both groups. This hypothesis is in agreement with “library” hypothesis that suggests the co-existence of different variants of centromere tandem repeats, that over time undergo through expansion and shrinkage and eventually the replacement of the most abundant variant with a different variant [52].

In conclusion, we demonstrated that the reverse transcriptase part of Ty3/gypsy centromeric retrotransposon (RT-CR) is highly conservative in segmental hexaploid *Th*. *intermedium* (J^r^J^vs^St) and its possible diploid progenitors but the abundance of the Ty3/gypsy CR varied to a large extend. Our results may provide valuable information to shed more light on the evolution and speciation in Triticeae.

## Materials and Methods

### Plant material

The following accessions were used in the experiments: *Th*. *intermedium* (PI 401200), *Th*. *bessarabicum* (PI 531711), *D*. *villosum* (W6 21717), *P*. *spicata* (PI 635993) obtained from Germplasm Research International Network; common wheat cv. Ivolga and cv. Tulaykovskaya 100 with 6J^r^(6D) substitution [51].

### DNA extraction, PCR amplification, cloning and sequencing

DNA was isolated from young leaves or seedlings according to Bernatzky and Tanksley (1986) [53]. The primers TAID_F 5’-TGGTACTTGGCGTCTGTGTG-3’ and TAID_R 5’-CTCGATTCCCTGTGGAGTA-3’ were designed based on conservative nucleic acid sequence of the reverse transcriptase gene of the CRM retrotransposon. Each PCR mixture (25 μl) contained 0.5 μl of DNA (50–300 ng) solution, 0.5 μl of each primer (10 pM/μL), 200 μM of each dNTP, 2.5 μM MgCl_2_, 2.5 μl of 10×Taq buffer, and 0.5 U of Taq polymerase. The PCR was performed at the following conditions: 5 min at 95°C; 35 cycles: 30 s at 95°C, 30 s at 58°C, 30 s at 72°C; 10 min at 72°C. The PCR products obtained in all analyzed species were run on agarose gel, and one distinct band approximately 500 bp was observed.

The primers WxF3 (5-TCTGGTCACGTCCCAGCTCGCCACCT-3) and WxVT1R (5-ACCCCGCGCTTGTAGCAGTGGAAGT-3) [54] were used for amplification and sequence exon I *GBSS*I gene and from species *Th*. *bessarabicum* (PI 531711), *D*. *villosum* (W6 21717), *P*. *spicata* (PI 635993) (S4 Fig). Each PCR mixture (25 μl) contained 1.0 μl of DNA (50–300 ng) solution, 1.0 μl of each primer (10 pM/μL), 200 μM of each dNTP, 2.5 μM MgCl_2_, 2.5 μl of 10×Taq buffer, and 0.5 U of Taq polymerase. The PCR was performed at the following conditions: 5 min at 95°C; 40 cycles: 30 s at 95°C, 60 s at 64°C, 120 s at 72°C; 10 min at 72°C.

The amplified products were cloned into pGEMT®-Easy Vector in *E*. *coli* strain DH10B. The individual clones were sequenced using ABI 3130xl Genetic Analyzer (Applied Biosystems, Foster City, CA, USA) according to the manufacturer’s recommendations.

### Sequence analysis and phylogenetic relationships

Alignment and translation of the sequences was performed using Mega6 software [55]. A search for homologous sequences was performed with a BLAST search (http://blast.ncbi.nlm.nih.gov). A search for conserved domains within a coding nucleotide sequences was performed using NCBI's CD-Search (http://ncbi.nlm.nih.gov/Structure/cdd/wrpsb.cgi).

Nucleic acid sequences of our RT-CRs were compared with 964 DNA sequences found using NCBI BLAST search (Nucleotide collection) by Mega6 software. The evolutionary history was inferred by using the Maximum Likelihood method based on the Tamura 3-parameter model. The bootstrap consensus tree inferred from 1000 replicates [55]. The sequences were analyzed without primer regions.

Deduced amino acid sequences of our RT-CRs were compared with the RT domains of known centromeric repeats of rice (DQ458289.1), maize (AAM94350.1), wheat (ABI96971.1) and barley (AAK94517.1) by Mega6 software. The dendrogram was constructed using the Maximum Likelihood method based on the JTT matrix-based model. The bootstrap consensus tree inferred from 1000 replicates [55]. The RT-CR element of *A*. *thaliana* (AAF79348.1) was used as an out-group.

### *In situ* hybridization

Chromosome preparations were carried out as described in [56].

The PCR products obtained from *Th*. *bessarabicum* and *Th*. *intermedium* were labeled with biotin-16-dUTP, from *P*. *spicata* with digoxigenin-11-dUTP, from *D*. *villosum* with biotin-16-dUTP or digoxigenin-11-dUTP by PCR according to the manufacturer’s instructions (Roche, Germany); the probes were designated *Thbe*, *Thin*, *Pssp*, and *Davi*, respectively. One- and multicolor FISH experiments were performed as described in Karlov et al. (2003) [57]. The chromosomes were counterstained with 1 mg/ml DAPI and mounted in Vectashield (Vector laboratories, UK).

After post-hybridization washing of *Th*. *intermedium* chromosome mitotic preparations as described in Komuro et al. [58] multicolor genomic *in situ* hybridization (mcGISH) was conducted on the same slides as described in Kishii et al. [23] with modifications described in Salina et al. [51]. The probes were the *P*. *spicata* (St genome) and *D*. *villosum* (V genome) genomic DNA (50 ng/preparation) labeled with digoxigenin-11-dUTP and with biotin-16-dUTP, respectively, by nick translation according to the manufacturer’s instructions (Roche, Germany). The chromosomes were counterstained with 1 mg/ml DAPI and mounted in Vectashild (Vector laboratories, UK).

An AxioImager M1 fluorescent microscope (Zeiss) was used to observe chromosome preparations. The metaphase plates with fluorescent signals were photographed with a monochrome AxioCam MRm CCD camera and visualized using Axiovision software (Zeiss).

### Quantitative PCR

To estimate relative quantity of RT-CR sequence in the genomes of the studied species qPCR assay was performed according to Yaakov et al. (2013). Each template for qPCR analysis was run in triplicate reactions (technical replicates), each consisting of 2.5 μL of reaction mix containing SYBR Green I + ROX (Syntol LTD), 0.78, 1.56, 2.00, 3.12, 6.25, 12.50, 25.00 ng of DNA template, 1.0 μL of each forward and reverse primer (10 pM/μL). The relative quantity (RQ) of RT-CR sequences was calculated according to Yaakov et al. [37]; each reaction was compared to amplification of the *GBSS*I gene, as we suppose that it is found in one copy in each genome [39, 54, 59–61]. Primers for *GBSS*I gene sequence were developed on the basis of multiple alignment of the exon 1 sequences for species *Th*. *bessarabicum*, *P*. *spicata*, *D*. *villosum* received by us and validated using conventional PCR followed by gel electrophoresis (S1 Table, S4 and S5 Figs). The qPCR was performed at the following conditions: 10 min at 95°C, then 40 cycles of 15 s at 95°C and 20 s at 58°C and 30 s at 72°C. The normalized quantities were then compared to the quantity in *D*. *villosum* (W6 21717) as it showed the least quantity of RT-CR sequence.

## Supporting Information

S1 Fig Multiple alignment of the RT-CR sequences. (TIF)

S2 Fig The dendrogram of the RT-CR DNA sequences and their homologs from the NCBI Nucleotide collection search database. The evolutionary history was inferred by using the Maximum Likelihood method based on the Tamura 3-parameter model. The bootstrap consensus tree inferred from 1000 replicates.
(PDF)

S3 Fig Sequential FISH and GISH of *T*. *aestivum* cv. Tulaykovskaya 100 with 6J^r^(6D) substitution [51]. a) Hybridization of the *Thin* probe on chromosomes of Tulaykovskaya 100. b) Identification of 6J^r^ chromosomes of *Th*. *intermedium* in Tulaykovskaya 100 by multicolor GISH with the labeled genomic DNA of *P*. *spicata* (green) and *D*. *villosum* (pink, signal absent) (blocked with genomic DNA of *T*. *aestivum* cv. Ivolga). Chromosomes counterstained with DAPI (blue). Chromosome 6J^r^ shown with asterisk. Bar = 10 μm.
(TIF)

S4 Fig Multiple sequence alignment of partial exon 1 sequences of *GBSS*I orthologs obtained using primers WxF3/WxVT1R [58] with the samples of *Th*. *bessarabicum* (PI 531711), *D*. *villosum* (W6 21717), *P*. *spicata* (PI 635993). The positions of primers DwaxyQ used in qPCR analysis are indicated in arrows and color highlighted.
(JPG)

S5 Fig PCR validation primers DwaxyQ used in qPCR analyses. **As a negative control (NC), water was served as a template in the PCR reaction.** A 100 bp size-ladder was used.
(JPG)

S1 Table List of primer sequences used for qPCR amplifications. (DOCX)
