# Supplementary figures and images for: Variation in Copy Number of Ty3/Gypsy Centromeric Retrotransposons in the Genomes of Thinopyrum intermedium and Its Diploid Progenitors
Source: PLoS One. 2016 Apr 27;11(4):e0154241. doi: 10.1371/journal.pone.0154241 (PMC4847875; doi:10.1371/journal.pone.0154241)

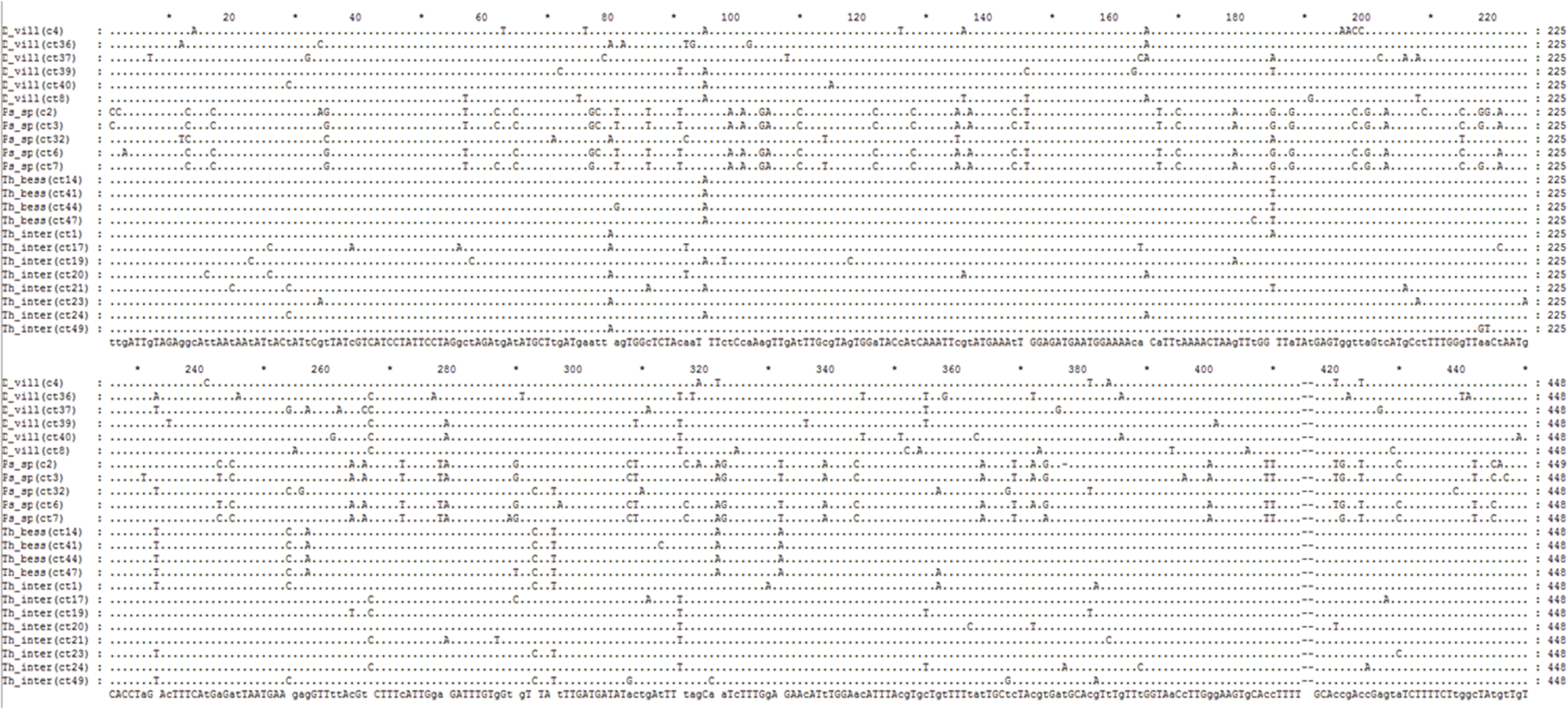

Supplement: S1 Fig — (TIF) [file pone.0154241.s001.tif]

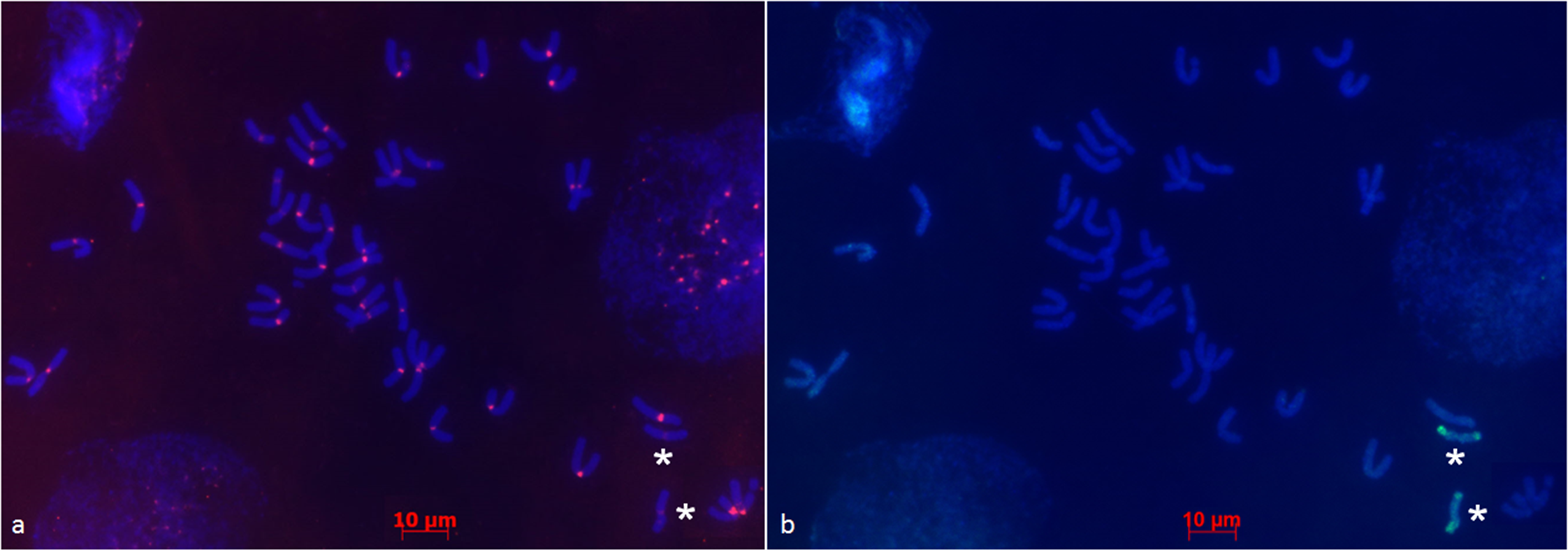

Supplement: S3 Fig — a) Hybridization of the Thin probe on chromosomes of Tulaykovskaya 100. b) Identification of 6Jr chromosomes of Th. intermedium in Tulaykovskaya 100 by multicolor GISH with the labeled genomic DNA of P. spicata (green) and D. villosum (pink, signal absent) (blocked with genomic DNA of T. aestivum cv. Ivolga). Chromosomes counterstained with DAPI (blue). Chromosome 6Jr shown with asterisk. Bar = 10 μm. (TIF) [file pone.0154241.s003.tif]

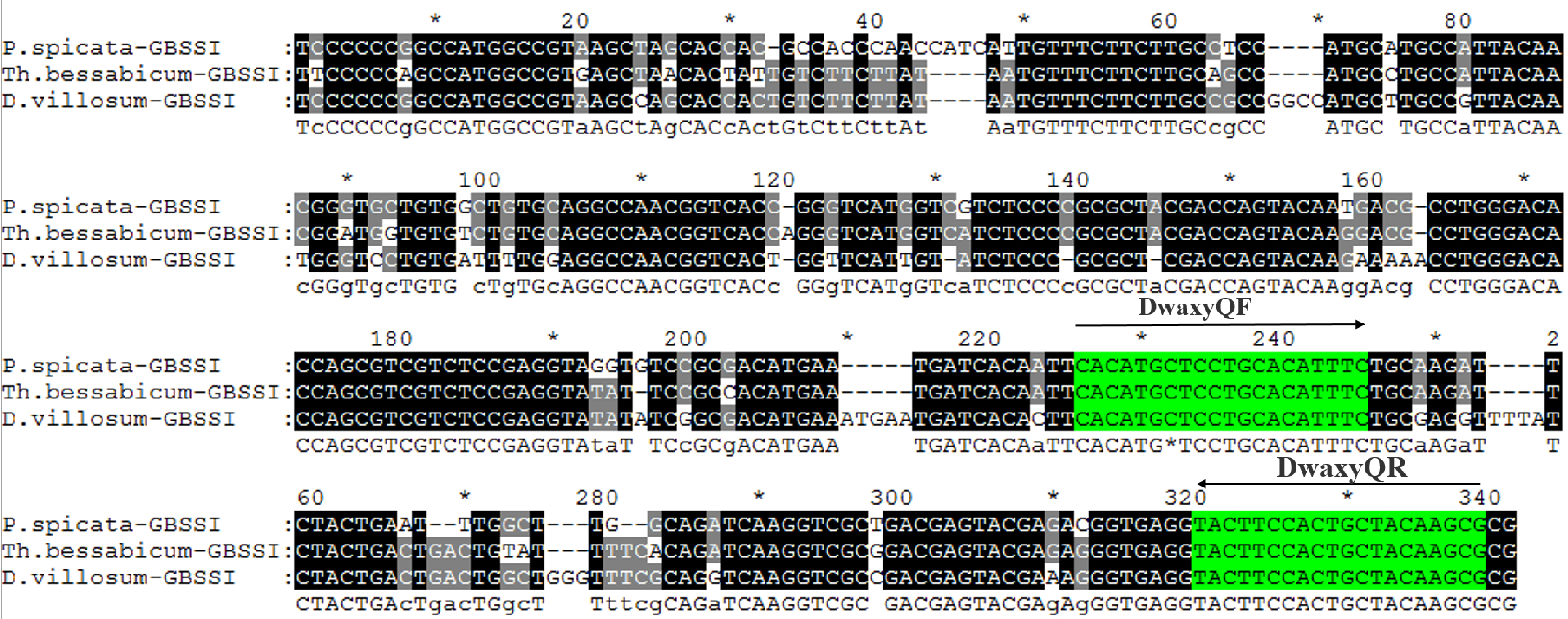

Supplement: S4 Fig — The positions of primers DwaxyQ used in qPCR analysis are indicated in arrows and color highlighted. (JPG) [file pone.0154241.s004.jpg]

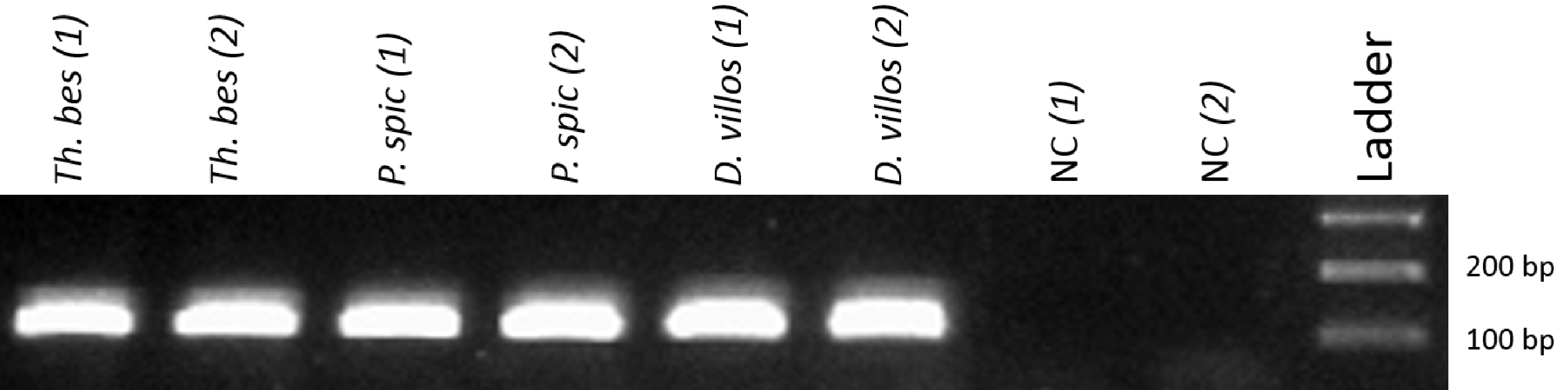

Supplement: S5 Fig — As a negative control (NC), water was served as a template in the PCR reaction. A 100 bp size-ladder was used. (JPG) [file pone.0154241.s005.jpg]
