# Supplementary material for: Variation in Copy Number of Ty3/Gypsy Centromeric Retrotransposons in the Genomes of Thinopyrum intermedium and Its Diploid Progenitors
Source: PLoS One. 2016 Apr 27;11(4):e0154241. doi: 10.1371/journal.pone.0154241 (PMC4847875; doi:10.1371/journal.pone.0154241)
Supplement: S1 Table — (DOCX) [file pone.0154241.s006.docx]

**TABLE**

**List of primer sequences used for qPCR amplifications**

| Amplified sequence/  Gene | Primer name | Forward primer | Reverse primer | Species | Amplicon length, bp | Efficiencies | R^2^ |
| --- | --- | --- | --- | --- | --- | --- | --- |
| RT-CR | TAID | 5'-TGGTACTTGGCGTCTGTGTG-3' | 5'-CCTCGATTCCCTGTGGAGTA-3' | *Th. bessarabicum* | 487 | 97,3 | 0,9974 |
|  |  |  |  | *P. spicata* | 487 | 102,2 | 0,999 |
|  |  |  |  | *D. villosum* | 487 | 97,8 | 0,9891 |
| GBSSI | DwaxyQ | 5'-CACATGCTCCTGCACATTTC-3' | 5'-CGCTTGTAGCAGTGGAAGTA-3' | *Th. bessarabicum* | 103 | 101,6 | 0,9971 |
|  |  |  |  | *P. spicata* | 107 | 102,3 | 0,9985 |
|  |  |  |  | *D. villosum* | 114 | 104,5 | 0,9921 |
